# Perinatal health in a cohort of children conceived after assisted reproduction in the UK: a population-based record-linkage study

**DOI:** 10.1136/bmjopen-2024-091910

**Published:** 2024-11-11

**Authors:** Mitana Purkayastha, Alastair Sutcliffe, Daniel R Brison, Scott M Nelson, Deborah Lawlor, Stephen A Roberts

**Affiliations:** 1 UCL GOS ICH, London, UK; 2 Division of Developmental Biology & Medicine, The University of Manchester, Manchester, UK; 3 School of Medicine, Dentistry & Nursing, University of Glasgow, Glasgow, UK; 4 NIHR Bristol Biomedical Research Centre, Bristol, UK; 5 MRC Integrative Epidemiology Unit, University of Bristol, Bristol, UK; 6 Division of Population Health, Health Services Research & Primary Care, The University of Manchester, Manchester, UK

**Keywords:** PERINATOLOGY, EPIDEMIOLOGY, REPRODUCTIVE MEDICINE

## Abstract

**Abstract:**

**Objective:**

To compare the risk of hospitalisation for conditions originating in the perinatal period between children conceived via assisted reproductive technology and those that are naturally conceived, differentiating by treatment type.

**Study design, setting and participants:**

Population-based record-linkage study of children born after assisted reproduction in the UK between 2002 and 2009 (n=44 618), their naturally conceived siblings (n=8462) and matched naturally conceived population (n=89 072) controls linked to their hospital inpatient records up to 31 March 2016.

**Primary and secondary outcome measures:**

Robust estimates of the overall and cause-specific risk of hospital admission for adverse perinatal events and the comparison of outcomes by type of treatment.

**Results:**

Over the study period, 17 132 (38.40%) children conceived via assisted reproduction and 30 306 (34.02%) and 1738 (20.54%) naturally conceived population and sibling controls, respectively, were admitted to the hospital for severe perinatal events. Compared with the population controls, singletons (Risk ratio (95% CI 1.30 (1.26, 1.34))) and twins (1.01 (0.99, 1.03)) conceived via assisted reproduction exhibited a higher risk of hospitalisation for any adverse perinatal event. However, no such increase was observed in the within-sibling analysis (0.97 (0.84, 1.12)). Similar patterns were seen for diagnoses related to length of gestation and fetal growth (vs population controls: 1.37 (1.29, 1.46); vs siblings: 1.17 (0.86, 1.60)); birth trauma (vs population controls: 1.23 (1.04, 1.44); vs siblings: 0.78 (0.47, 1.30)); respiratory and cardiovascular disorders (vs population controls: 1.28 (1.20, 1.38); vs siblings: 0.72 (0.53, 0.98)); infections (vs population controls: 1.30 (1.06, 1.59); vs siblings: 0,68 (0.24, 1.90)) and several other conditions. Associations were similar when comparing in vitro fertilisation to intracytoplasmic sperm injection and were higher when comparing fresh to frozen embryo transfers.

**Conclusion:**

Children conceived via assisted reproduction showed modest increases in the risk of hospitalisations for severe perinatal events when compared with population controls, although these findings were attenuated in the sibling analyses. The imprecision of within-sibling analyses highlights the need for larger studies to explore potential causal effects.

**STRENGTHS AND LIMITATIONS OF THIS STUDY**

- Meticulous linkage of robust, routinely collected administrative health data to yield a large cohort that is nationally unique, thus increasing the generalisability, accuracy and precision of results from subsequent analyses.
- Linkage to the hospital admissions and outpatient database provides long-term mortality and morbidity outcome data on offspring for use in longitudinal research, policy planning and strategic development.
- Identification of naturally conceived siblings as well as matched naturally conceived population controls allows exploration of the association of assisted reproductive technology (ART) with adverse offspring outcomes while accounting for parental factors related to subfertility, which may confound these associations.
- Comparison of findings between the two approaches (ART vs naturally conceived population controls and ART vs naturally conceived siblings) mentioned above increases confidence in findings.
- The validity of the cohort was tested by means of an exemplar analysis.

## Introduction

 The use of assisted reproductive technology (ART) has risen dramatically over the last five decades, with more than 9 million children conceived via ART globally.^1^ In the UK, 2.9% of all births in 2018 were as a result of ART.^2^ Despite this widespread adoption, a primary concern among the families of ART children is whether their offspring are at an increased risk of adverse health outcomes, particularly in the perinatal period. It is well known that ART pregnancies are associated with a higher risk of preterm birth (PTB) and low birth weight (LBW) compared with naturally conceived (NC) pregnancies.^3 4^ Although this was initially thought to be driven by the higher rates of twin or other multiple pregnancies related to the transfer of two or more embryos in ART, more recent evidence shows that even ART singletons exhibit higher risks of PTB; small for gestational age (SGA) or LBW; perinatal/neonatal mortality and admission to neonatal intensive care units when compared with NC children.^35^,^11^

Potential drivers of this increased risk include factors associated with the ART procedure itself and/or those causing or contributing to the underlying subfertility.^12^ Previous attempts to delineate the relative contributions have been conflicting, with a UK study (n=1 44 018) reporting that the risk of PTB and LBW was increased if oocyte donation was required while the risk of macrosomia increased with advancing maternal age and a history of previous live births.^4^ Furthermore, infertility as a consequence of cervical problems increased the odds of all three outcomes—PTB, LBW and macrosomia.^4^ A Finnish study (n=65 723) using administrative registers compared ART children to their NC siblings and found that the increased risk of adverse perinatal outcomes could largely be attributed to factors other than the ART procedure itself.^13^ However, direct evidence from within-ART studies also suggests that factors in the ART process itself have an impact on birth outcomes, with retrospective and prospective randomised trials showing that the composition of the embryo culture medium is associated with altered birth weight and child growth in ART offspring.^14^,^18^ Furthermore, embryo freezing has also been shown to be associated with these outcomes, including in comparisons of fresh frozen transfer siblings from the same couple.^19^,^21^ Similarly, an Australian cohort study (n=5469) found that singleton births from in vitro fertilisation (IVF) were associated with LBW, PTB and neonatal death to a greater extent than births from intracytoplasmic sperm injection (ICSI), while frozen embryo transfers (ETs) appeared to eliminate all significant adverse outcomes associated with ICSI but not with IVF.^22^

The inability to distinguish between the contribution of ART treatment factors and parental subfertility to adverse perinatal outcomes can be addressed to a certain extent by prospective cohorts including control populations of NC children born to parents with established subfertility (different from infertility in terms of the time of unwanted non-conception)^23^ or through within-sibling analyses (where comparisons are made between ART and their NC siblings or ART siblings born from fresh and frozen ETs) to better control for factors related to subfertility and other family confounders under the assumption that these parental factors would be the same (or very similar) within sibling groups.^24^ Several electronic health record linkage studies have used sibling analyses, with some reporting lower mean birth weight, shorter gestational duration and/or increased risk of SGA and PTB on comparison of ART and NC children and others finding that the associations observed in the (unrelated) population were attenuated in the sibling analyses.^1325^,^27^ However, these studies have been relatively small with the number of discordant sibling groups ranging between 1245 and 6458. The Committee of Nordic ART and Safety (CoNARTaS) recently used the largest electronic health record cohort (n=4 510 790 singleton deliveries, including 33 056 discordant sibling groups) to show that, compared with the NC population, ART conception with fresh and frozen ETs increased the risk of SGA and LGA, respectively.^28^ Furthermore, both types of ETs increased the risk of PTB and these findings were consistent in the population and within-sibling analyses.^28^ This was also in agreement with previous evidence from the UK cohort used here (63 877 ART-conceived children, 11,343 NC siblings and 127 544 unrelated NC children) where the comparison with NC siblings and unrelated controls showed that fresh and frozen ETs were associated with lower and higher mean birth weight, respectively.^29^ Separate analyses of the CoNARTaS cohort also demonstrated that previous within-sibling analyses that reported an apparent protective effect of ART conception on perinatal mortality using smaller study samples were biased by the combination of selective fertility (ie, increased likelihood of becoming pregnant following perinatal mortality in a given time period compared with those with a healthy live birth) and carryover (ie, treatment or outcome in the first sibling affecting treatment or outcome in subsequent siblings).^30^

The objective of the current study was to (1) compare the risk of development of severe perinatal complications and conditions requiring hospitalisation during the first 7 days of life in children conceived via ART to those that are NC and (2) assess whether these associations differed by the type of ART treatment (IVF vs ICSI, embryo cryopreservation).^17^ This adds to previous studies using population and sibling analyses by focusing on more severe perinatal outcomes that require hospital admission and exploring risk by carrying out ART versus NC as well as within-ART comparisons.

## Methods

### Original study cohort

Children born to women who had undergone ART in the UK between 1 April 1997 and 31 July 2009, their NC siblings (NCS), and two NC population controls (NCP) per ART child matched for age, sex and multiplicity were identified through a one-off linkage between the Human Fertilisation & Embryology Authority (HFEA) register and the Office for National Statistics birth registration dataset. All three study groups were then linked to their health outcome data up to 31 March 2016 using the Hospital Episode Statistics (HES) dataset containing details of all admissions at NHS hospitals in England.^29^ A subgroup of ART children with NCS (sART) was also created for the within-sibling analysis to allow comparison under the assumption that parental factors would be the same (or very similar) within sibling groups. An overview of the linkage methodology has been provided in online supplemental figures 1 and 2 and further details have been reported previously.^29^

### Study inclusion and exclusion criteria

**Figure 1:**
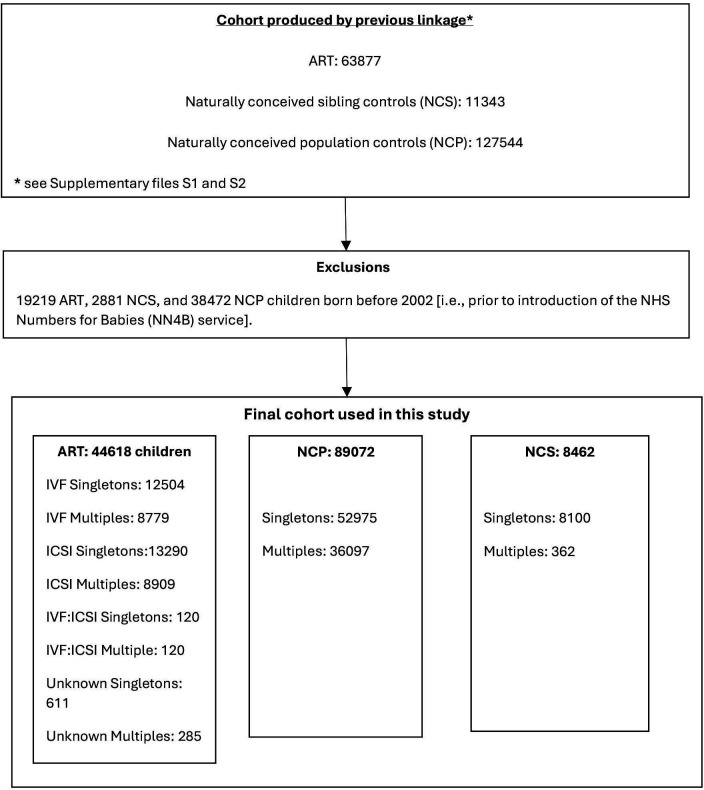
Flowchart showing creation of study cohort. ART, assisted reproductive technology; ICSI, intracytoplasmic sperm injection; IVF, in vitro fertilisation; NCP, naturally conceived population controls; NCS, naturally conceived siblings .

The current study focused on a subgroup of the original study cohort born between 2002 and 2009 only (study flowchart shown in figure 1). A lower limit of 2002 was applied as the NHS Numbers for Babies (NN4B) service was introduced in this year, allowing maternity staff in England to request an NHS number for babies in the hospital after birth using an online system as part of the Statutory Birth Notification process. Prior to this, babies were allocated NHS numbers by registrars at birth registration which could take up to 6 weeks. Previous studies exploring the impact of changes to data collection over time on coverage and completeness of linked follow-up records for children reported unreliable linkages between births and follow-up records before 2002, evidenced by underestimation of mortality and hospital readmission rates.^31 32^ An upper limit of 2009 was applied in keeping with HFEA legislation. Consent for disclosure of information for research was not collected from patients who underwent treatment at a licensed fertility clinic prior to September 2009 (although consent could be withdrawn retrospectively), thus permitting linkage of these individuals (and any children born thereafter) to other datasets. However, since 1 October 2009, prospective consent for research use of data has been made mandatory, and low overall consent rates which varied between fertility clinics have cast doubt on the validity of research conducted using HFEA register data recorded after this date.^33 34^

The study exclusion criteria were as follows: (a) ART children born to women who permanently lived outside the UK but travelled to the UK for treatment; (b) ART children conceived in the UK but born outside of England, Wales and Scotland; (c) siblings born outside of England, Wales and Scotland; (d) siblings born outside of the study period (as their conception status could not be verified); (e) cases that had withdrawn consent for their data to be used for research and (f) children born after donor ART, in keeping with HFEA statutes preventing the viewing of identifiable data relating to these children by any third party. Triplets and higher-order births were excluded from the analysis as they are known to be associated with adverse outcomes such as higher infant mortality, birth defects, premature birth and low birth weight.^35^

Ethical approval and waiver of the requirement for individual consent were obtained from the UK Health Research Authority Confidentiality Advisory Group and the London Research Ethics Committee—Hampstead (references ECC 4-03(g)/2012 and 12/LO/1063, respectively).

### Outcome data

A perinatal event was defined as hospital admission for a primary diagnosis corresponding to Chapter XVI (“Certain conditions originating in the perinatal period”; COPP) of the ICD-10 (International classification of diseases, 10th revision) classification (online supplemental table S3) within 15 days after birth. The ICD-10 defines the ‘perinatal period’ as the time period starting at 22 completed weeks (154 days) of gestation and lasting through 7 days after birth. For this study, this definition was extended to 15 days after birth to allow for late hospital recording.

The primary outcome measure was the risk of hospital admission for any perinatal event and by individual perinatal diagnosis groups. The secondary analysis explored whether this risk varied by the type of ART treatment (ie, IVF vs ICSI, fresh vs frozen ETs). Only inpatient contacts were included to allow examination of diagnoses on the more severe end of the spectrum requiring hospitalisation. Moreover, the HES outpatient clinic dataset is only available for linkage from 2003, preventing exploration of any contacts prior to this.

### Statistical analyses

Binary variables for the first occurrence of any perinatal event and diagnostic group-specific events were created, and generalised linear models with a log link function were used to estimate risk ratios (RRs) and 95% CIs for each outcome for the ART versus NCP and sART versus NCS comparisons separately. Separate analyses were conducted for singletons and twins. The ART versus NCP model was adjusted for maternal age at delivery (grouped into 25–29, 30–34, 35–39, 40–44 and ≥45 years); year of birth; socioeconomic status (deciles of the UK census-derived Index of Multiple Deprivation (IMD), the official measure of relative deprivation for small areas or neighbourhoods in the UK)^36^ at the time of first hospital admission; sex and ethnicity (grouped into White/non-White).

For comparisons of sART versus NCS, a maternal ID cluster was used to create family-matched models adjusted for year of birth; maternal age at delivery; sex and order of pregnancy (grouped into first, second and >2) to allow for within-family correlations. IMD and ethnicity were not included as the underlying effects they represent would have remained constant within families.

Further models explored the effects of ART subgroups (IVF/ICSI and fresh/frozen ETs), with each subgroup being compared with the NCP cohort to estimate RRs. Within-subtype analyses were also carried out comparing IVF versus ICSI and fresh versus frozen ETs. Due to small numbers, these analyses were not performed in the sibling cohort. All statistical analyses were performed using the statistical software package STATA V.16.0.

### Patient and public involvement

No patients were involved. Due to the very personal nature of the treatments involved, it was not appropriate to contact the families directly, thus preventing us from involving patients or the public in the design, conduct, reporting or dissemination plans of our research. However, we carried out an a priori investigation (assisted by the Royal College of Obstetrics and Gynaecology Women’s Health panel and Infertility UK) to identify the primary concerns of mothers with ART-conceived children. This work demonstrated that the families of ART children had ‘unmet information needs’ about the impact of assisted conception on their child’s future health.^37^

## Results

### Characteristics of the study population

The study cohort comprised of 44 618 ART children, 8462 NCS (siblings of 8318 ART children (sART)) and 89 072 matched NCP controls born between 2002 and 2009 (table 1). Of these, 17 132 (38.40%) ART, 30 306 (34.02%) NCP and 1738 (20.54%) NCS children were admitted to the hospital for COPP.

**Table 1:** Demographic characteristics of study cohort (2002–2009)

Infants	ART	NCP	sART	NCS
	44 618	89 072	8318	8462
Sex				
Female	22 078 (49.48%)	44 092 (49.50%)	4017 (48.29%)	4161 (49.17%)
Male	22 540 (50.52%)	44 980 (50.50%)	4301 (51.71%)	4301 (50.83%)
Multiplicity				
Singleton children	26 525 (59.45%)	52 975 (59.47%)	5686 (68.36%)	8100 (95.72%)
Multiple children	18 093 (40.55%)	36 097 (40.53%)	2632 (31.64%)	362 (4.28%)
IMD decile at earliest appointment				
1 (most deprived)	1474 (3.30%)	9218 (10.35%)	242 (2.91%)	214 (2.53%)
2	1939 (4.35%)	8054 (9.04%)	321 (3.86%)	291 (3.44%)
3	2385 (5.35%)	7100 (7.97%)	377 (4.53%)	351 (4.15%)
4	2841 (6.37%)	6819 (7.66%)	447 (5.37%)	462 (5.46%)
5	3304 (7.41%)	6322 (7.10%)	582 (7.00%)	552 (6.52%)
6	3740 (8.38%)	6037 (6.78%)	713 (8.57%)	676 (7.99%)
7	4282 (9.60%)	6078 (6.82%)	808 (9.71%)	758 (8.96%)
8	4606 (10.32%)	5985 (6.72%)	856 (10.29%)	860 (10.16%)
9	5224 (11.71%)	6083 (6.83%)	1033 (12.42%)	1022 (12.08%)
10 (least deprived)	5223 (11.71%)	5487 (6.16%)	1126 (13.54%)	1096 (12.95%)
Missing	9600 (21.52%)	21 889 (24.57%)	1813 (21.80%)	2180 (25.76%)
Year of birth				
2002	4980 (11.16%)	9933 (11.15%)	1170 (14.07%)	937 (11.07%)
2003	5379 (12.06%)	10 788 (12.11%)	1253 (15.06%)	1012 (11.96%)
2004	5559 (12.46%)	11 078 (12.44%)	1271 (15.28%)	1067 (12.61%)
2005	5662 (12.69%)	11 326 (12.72%)	1271 (15.28%)	1078 (12.74%)
2006	6275 (14.06%)	12 513 (14.05%)	1217 (14.63%)	1100 (13.00%)
2007	6342 (14.21%)	12 701 (14.26%)	1058 (12.72%)	1199 (14.17%)
2008	6347 (14.23%)	12 718 (14.28%)	670 (8.05%)	1260 (14.89%)
2009	4074 (9.13%)	8015 (9.00%)	408 (4.91%)	809 (9.56%)
Ethnicity				
White	43 330 (97.11%)	85 242 (95.70%)	8094 (97.31%)	8279 (97.84%)
Non-white	1288 (2.89%)	3830 (4.30%)	224 (2.69%)	183 (2.16%)
Maternal age at delivery				
≤25	490 (1.10%)	19 770 (22.20%)	151 (1.82%)	77 (0.91%)
25–29	3297 (7.39%)	17 054 (19.15%)	686 (8.25%)	462 (5.46%)
30–34	14 393 (32.26%)	26 489 (29.74%)	2948 (35.44%)	2213 (26.15%)
35–39	19 791 (44.36%)	18 899 (21.22%)	3608 (43.38%)	4031 (47.64%)
≥40	6643 (14.89%)	5523 (6.20%)	924 (11.11%)	1679 (19.84%)
Missing	4 (0.01%)	1337 (1.50%)	1 (0.01%)	0 (0.00%)

ART, assisted reproductive technology; IMD, Index of Multiple DeprivationNCP, naturally conceived population controls; NCS, naturally conceived siblings; sART, ART children with NC siblings

### Primary analysis: risk of hospital admission for any and diagnosis-specific perinatal events

The absolute risk of any perinatal event and specific perinatal diagnoses for each cohort has been shown in online supplemental table S4.

**Figure 2:**
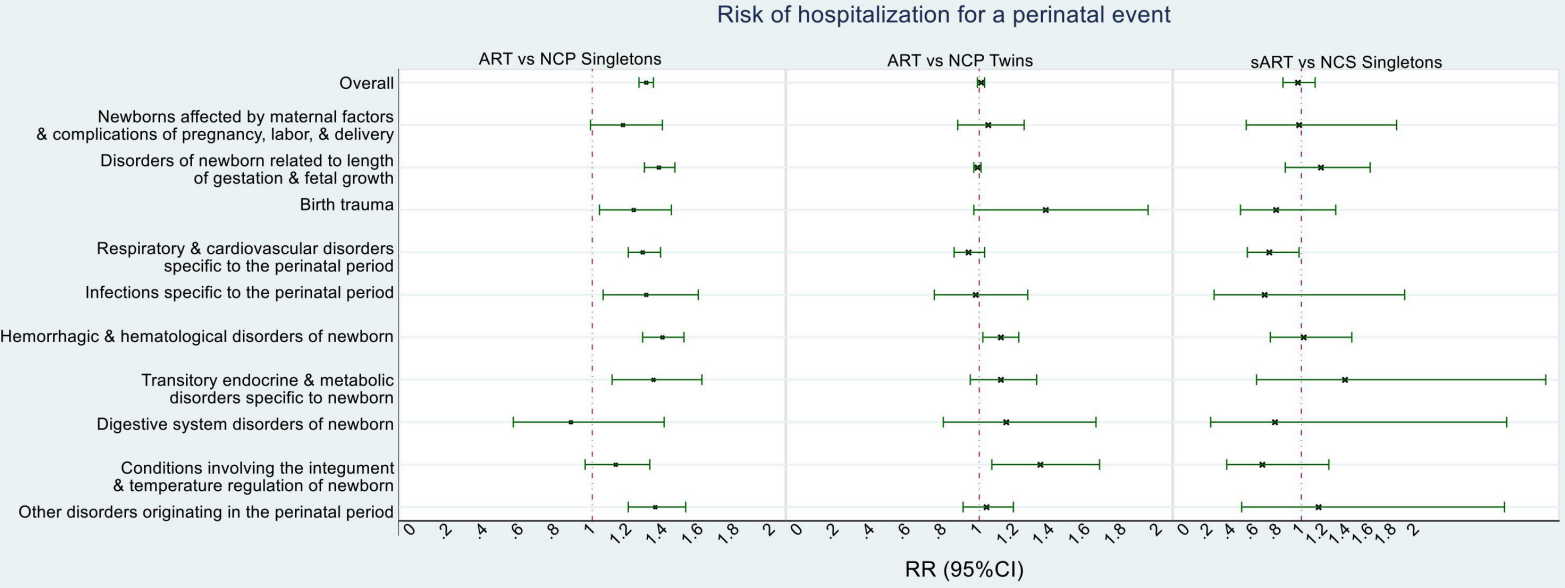
Risk of hospitalisation for a perinatal event. ART, assisted reproductive technology; NCR, naturally conceived population controls; NCS, naturally conceived siblings; RR, risk ratio.; sART, ART children with NC siblings.

Both ART singletons (RR 1.30, 95% CI 1.26, 1.34) and ART twins (RR 1.01, 95% CI 0.99, 1.03) exhibited a higher risk of hospital admission for any COPP when compared with the corresponding matched NCP subcohorts (figure 2 and online supplemental table S5). However, no such increase was observed in the sART versus NCS comparison (RR 0.97, 95% CI 0.84, 1.12).

ART singletons exhibited higher risk of hospital admission for adverse outcomes related to length of gestation and fetal growth (RR 1.37, 95% CI 1.29, 1.46); birth trauma (RR 1.23, 95% CI 1.04, 1.44); respiratory and cardiovascular disorders (RR 1.28, 95% CI 1.20, 1.38); infections (RR 1.30, 95% CI 1.06, 1.59); haemorrhagic and haematological disorders of newborn (RR 1.39, 95% CI 1.28, 1.51); transitory endocrine and metabolic disorders specific to newborn (RR 1.34, 95% CI 1.11, 1.61) and other disorders originating in the perinatal period when compared with NCP singletons (RR 1.35, 95% CI 1.20, 1.52; figure 2 and online supplemental table S5). However, the sART versus NCS analyses did not show statistically robust associations with any outcomes, although the estimates were imprecise with wide confidence intervals that overlapped with some of those seen in the ART versus NCP comparison (figure 2 and online supplemental table S5).

ART twins exhibited higher risk of hospital admission for haemorrhagic and haematological disorders of newborn (RR 1.12, 95% CI 1.02, 1.22) and conditions involving the integument and temperature regulation of newborn when compared with NCP twins (RR 1.34, 95% CI 1.07, 1.67; figure 1 and online supplemental table S5.

### Secondary analysis: risk of hospital admission by ART treatment type

#### Intracytoplasmic sperm injection (ICSI) versus in vitro fertilisation (IVF)

Compared with the matched NCP controls, children born after IVF with and without ICSI exhibited similar higher risk of hospital admissions for COPP (ICSI vs NCP RR 1.07, 95% CI 1.05, 1.09; IVF vs NCP RR 1.11, 95% CI 1.08, 1.13).

Furthermore, children born after ICSI had a somewhat lower risk of hospital admission for any COPP compared with those conceived via IVF without ICSI (RR 0.96, 95% CI 0.94, 0.98; table 2). Further analysis by diagnosis groups showed that children born after ICSI had a lower risk of hospital admission for disorders of newborn related to length of gestation and fetal growth (RR 0.91, 95% CI 0.88, 0.94) and a higher risk of respiratory and cardiovascular disorders (RR 1.10, 95% CI 1.03, 1.19) and transitory endocrine and metabolic disorders (RR 1.18, 95% CI 1.00, 1.40) when compared with children conceived via seen for IVF without ICSI (table 2).

**Table 2:** Event rate and risk of hospitalisation for any and diagnosis-specific perinatal events by ART treatment type

	IVF(n=21 283)	ICSI(n=22 199)	ICSI vs IVF	Fresh embryo transfer(n=38 964)	Frozen embryo transfer(n=5620)	Fresh vs frozen embryo transfer
	No. of events (%)	No. of events (%)	RR (95% CI)	No. of events (%)	No. of events (%)	RR (95% CI)
Any perinatal diagnosis	8287 (38.93%)	8442 (38.02%)	0.96 (0.94, 0.98)	15 228 (39.08%)	1888 (33.5%)	1.16 (1.12, 1.20)
New-borns affected by maternal factors and complications of pregnancy, labour and delivery	365 (3.47%)	326 (3.49%)	1.10 (0.95, 1.29)	468 (3.07%)	67 (3.55%)	0.96 (0.77, 1.20)
Disorders of newborn related to length of gestation and fetal growth	5754 (54.68%)	4921 (52.69%)	0.91 (0.88, 0.94)	8388 (55.08%)	829 (43.91%)	1.42 (1.34, 1.51)
Birth trauma	238 (2.26%)	199 (2.13%)	0.89 (0.74, 1.08)	329 (2.16%)	52 (2.75%)	0.91 (0.70, 1.19)
Respiratory and cardiovascular disorders specific to the perinatal period	1387 (13.18%)	1359 (14.55%)	1.10 (1.03, 1.19)	2011 (13.21%)	301 (15.94%)	0.99 (0.89, 1.10)
Infections specific to the perinatal period	157 (1.49%)	156 (1.67%)	1.12 (0.89, 1.40)	226 (1.48%)	46 (2.44%)	0.73 (0.55, 0.98)
Haemorrhagic and haematological disorders of newborn	1186 (11.27%)	1065 (11.40%)	0.99 (0.91, 1.08)	1671 (10.97%)	276 (14.62%)	0.87 (0.78, 0.98)
Transitory endocrine and metabolic disorders specific to newborn	261 (2.48%)	279 (2.99%)	1.18 (1.00, 1.40)	412 (2.71%)	52 (2.75%)	1.17 (0.90, 1.51)
Digestive system disorders of newborn	41 (0.39%)	42 (0.45%)	1.05 (0.68, 1.62)	67 (0.44%)	6 (0.32%)	1.38 (0.67, 2.87)
Conditions involving the integument and temperature regulation of newborn	260 (2.47%)	237 (2.54%)	1.01 (0.85, 1.21)	386 (2.53%)	46 (2.44%)	1.10 (0.84, 1.45)
Other disorders originating in the perinatal period	526 (5.00%)	453 (4.85%)	0.91 (0.80, 1.03)	804 (5.28)	113 (5.99%)	1.03 (0.86, 1.24)
Missing	349 (3.32%)	303 (3.24%)	–	466 (3.06%)	100 (5.30%)	–

ART, assisted reproductive technology; ICSI, intracytoplasmic sperm injection; IVF, in vitro fertilisationRR, risk ratio

#### Fresh versus frozen ETs

Compared with the NCP controls, children born via fresh ET exhibited a higher risk and those born after frozen ET exhibited a lower risk of hospital admission for COPP (fresh ET vs NCP RR 1.10, 95% CI 1.08, 1.12; frozen ET vs NCP RR 0.95, 95% CI 0.91, 0.98).

Moreover, children born after fresh ET had a higher risk of hospital admission for any COPP when compared with those born after frozen ET (RR 1.16, 95% CI 1.12, 1.20; table 2). Further analysis by diagnosis groups showed that children conceived via fresh ET had a higher risk of hospital admission for disorders of newborn related to length of gestation and fetal growth (RR 1.42, 95% CI 1.34, 1.51) and a lower risk of hospital admission for infections (RR 0.73, 95% CI 0.55, 0.98) and haemorrhagic and haematological disorders (RR 0.87, 95% CI 0.78, 0.98) compared with children conceived via frozen ET (table 2).

## Discussion

This nationwide longitudinal record-linkage study found that, compared with matched NCP controls, singletons conceived through ART exhibited a higher risk of hospital admission for COPP, particularly for adverse outcomes related to the length of gestation and fetal growth; birth trauma; respiratory and cardiovascular disorders; infections; haemorrhagic and haematological disorders of newborn; transitory endocrine and metabolic disorders specific to newborn and other disorders originating in the perinatal period. The magnitudes of the associations were modest, with a relative risk of 1.30 (95% CI 1.26, 1.34) for any admission and ranging from 1.23 (95% CI 1.04, 1.44) to 1.39 (95% CI 1.28, 1.51) for cause-specific associations. These findings agreed with a previous meta-analysis of 30 studies that reported an increased risk of PTB, LBW and SGA in ART singletons.^38^ Several cohort studies comparing older ART children (aged between 5 years and 18 years) to those conceived naturally also reported observing a higher risk of adverse cardiometabolic outcomes (including insulin resistance, higher blood pressure and increased body fat percentage) and higher velocity of weight gain in the former.^39^,^41^ When ART children were compared with their NCS, there was no strong statistical support for a difference, with the point estimate for any hospital admission being close to the null value. Although this might be interpreted as suggesting that confounding, including parental causes of infertility, may explain the observed population control association, we acknowledge that the sibling sample size was relatively small and that the wide CIs, particularly for specific conditions, meant we could not robustly claim these results were different to the ART–NCP results. Therefore, the findings of the within-family analysis must be interpreted with caution, and the differences in estimates for the individual diagnostic groups between the ART–NCP and sART–NCS comparisons warrant further exploration in studies with larger numbers of participants.

ART babies born from frozen ET showed reduced overall risk of hospital admissions for COPP when compared with those born from fresh ET, while being conceived via ICSI compared with IVF without ICSI had little impact. The associations were relatively small, suggesting relative increases of 7% to 10%. Analysis by diagnosis groups showed that children conceived via ICSI or frozen ET were at a lower risk of hospital admission for disorders related to the length of gestation and fetal growth. These findings were in agreement with a recent meta-analysis of 65 studies that examined the risk of adverse perinatal outcomes in ART children and observed a lower risk of PTB and LBW in ICSI versus IVF singletons.^42^An Australian study of 18 429 children conceived via ART also reported higher perinatal risks in children from couples with female factor infertility (mainly treated with IVF) compared with those from couples with male factor infertility (mainly treated with ICSI).^43^ The majority of female partners in couples undergoing ICSI tend to be reproductively healthy and it has been suggested that this could potentially have beneficial effects on the perinatal outcomes of the child.^42^ The same meta-analysis by Pinborg *et al*, (2013) also found that frozen ET singletons had a lower risk of PTB compared with those conceived via fresh ET, and this was supported by several other studies that reported a significantly lower risk of PTB, LBW and SGA and a higher risk of LGA in frozen versus fresh ET singletons.^1942^,^45^ These differences could potentially be attributed to hormonal dysregulation of the uterine environment following ovarian hyper-stimulation, resulting in impaired placental function and restricting fetal growth in fresh cycles.^46 47^ In contrast, pregnancies arising from frozen blastocyst transfers have been shown to demonstrate better uterine perfusion and larger placental volume, potentially leading to improved fetal growth when compared with fresh blastocyst transfers.^48^,^50^ Alternatively, changes in the developmental processes during the early embryo stages, induced by the cryopreservation techniques, which consequently affected the intrauterine growth potential may also lead to an increased risk of LGA in children conceived via frozen ET.^42^ However, as there were too few participants to undertake sibling analyses by the type of ART in the current study, it was not possible to disentangle whether the increased risk of adverse perinatal outcomes in the ART cohort could be attributed to the reproductive technology per se or factors related to inherent infertility.

### Strengths and weaknesses

The main strength of this study lies in the meticulous linkage of robust, routinely collected administrative health data to provide hospital admissions for conditions occurring in the perinatal period.^17^ The risk of selection bias is also minimised by the mandatory nature of reporting all ART cycles carried out in the UK to the HFEA.^26^ The inclusion of two control groups (NCP and NCS) facilitates extrapolation of effect sizes and risk estimates to the general ART population as well as exploration of the effects of family confounders such as genetic and behavioural factors related to infertility and socioeconomic background. The two comparator groups have different sources of bias, including residual family-level confounding in the population analyses and possible bias due to carry-over effects in the sibling comparisons. The latter refers to situations where the exposure in one sibling influences outcomes in the other.^30^ When this is combined with selective fertility, it can result in strong bias as has been observed in previous studies reporting within-sibling analyses suggesting that ART protects against perinatal mortality, despite within-sibling and conventional general population analyses in the same studies showing ART increases PTB and SGA.^30^ Thus, despite differences in bias, there is increased confidence in the findings where results from the two comparator groups are similar. As noted above, the statistical inefficiency of sibling analyses in general and the relatively small number of sibling groups in this study limit the inferences that can be drawn here.

The main limitations of this study include those related to the identification of the study cohort itself and the subsequent linkage to the HES database. The current study included children conceived via ART between 2002 and 2009 and this was largely influenced by the effects of the NN4B, introduced in 2002, and changes in HFEA legislation with regard to consent for disclosure of information for research in 2009. The resultant smaller sample reduced power in the sibling analyses, limited our ability to carry out within-sibling analyses by the type of ART and prevented the exploration of perinatal health outcomes in children born outside the study period. Rapid advances in ART technologies in the last 10 years, particularly greatly increased use of single ETs with a concomitant reduction in dizygotic twinning and a rise in the use of extended embryo culture to blastocyst stage prior to fresh transfer, emphasise the urgent need to continue to prospectively monitor the next cohort of children from 2010 onwards. Although some studies have explored the influence of these changes as well as others (eg, timelapse incubators, changes in stimulation protocols, etc), the lack of data post-2009 limited our ability to explore the effects of changes in ART techniques over time and draw potential inferences in relation to current ART conceptions.^51^

The current study only included inpatient contacts; however, this would likely have had a minimal effect on the findings as initial exploration showed that very few patients were diagnosed with perinatal events through the outpatient clinics. Moreover, although HES data have been used extensively for research purposes, there have been long-standing concerns regarding the quality, completeness and coverage of records within health services and the academic community.^52^ Another limitation was that the method of definition of NC siblings used would be very sensitive to any linkage errors, with missed second ART babies appearing as conventional siblings. As a result, extensive quality assurance procedures were carried out on the linkage process to minimise this (see details in Purkayastha *et al*). Some cohort participants would not have HES records as they may have sought privately commissioned health treatment or their records were unavailable due to record-keeping error, coding error, linkage error or had been removed as a result of ethico-legal filtering (eg, where selected patients’ records are removed from extracts as they have registered an objection to their records being used for this purpose).^29^ However, approximately 98%–99% of hospital activity in England is estimated to be funded by the NHS, and the HES admitted patient care database covers all births in NHS hospitals, representing approximately 97·3% of births in England, thus making the creation of nationally representative cohorts possible.^19^ Consequently, we believe that the cohort will capture the vast majority of outcomes in couples who became pregnant. Finally, the weak/inaccurate identifier data on the HFEA register and the high threshold used for matching also meant that approximately 23% of children were lost during the linkage process; however, although unavailable for the study period explored here, future studies may be able to avoid this loss to follow-up as the HFEA now records both the mother’s and child’s NHS numbers on their register. Nevertheless, the findings of this study provide vital insight into the health of children born after ART, potentially facilitating the identification of at-risk individuals/families; contextualisation of levels and trends of disease burden and hospitalisation; informing affected patients and their families and also existing health services; allow for wider health system resource planning and anticipation of future health resource needs and contribute to the development of effective care pathways.

### Conclusion

The current study showed modest increases in the risk of hospital admissions for any COPP among ART-conceived children when compared with NCP controls, with attenuation to the null of these findings within siblings. However, the findings of the latter should be interpreted with caution due to limited power for the sibling analyses. Analysis by treatment type showed that frozen ET was associated with a reduced risk compared with those born from fresh ET, while being conceived via ICSI compared with IVF without ICSI had little impact. We acknowledge that limited power in our sibling analyses prevents robust interpretation and also highlights the need for larger studies exploring hospital admissions in the perinatal period.

## supplementary material

10.1136/bmjopen-2024-091910 online supplemental file 1

## Data Availability

Data may be obtained from a third party and are not publicly available.
