# Supplementary material for: Perinatal health in a cohort of children conceived after assisted reproduction in the UK: a population-based record-linkage study
Source: BMJ Open. 2024 Nov 11;14(11):e091910. doi: 10.1136/bmjopen-2024-091910 (PMC11555099; doi:10.1136/bmjopen-2024-091910)
Supplement: online supplemental file 1 [file bmjopen-14-11-s001.pdf]

Supplementary files

Supplementary file S1: Stage 1 of linkage (HFEA-ONS)

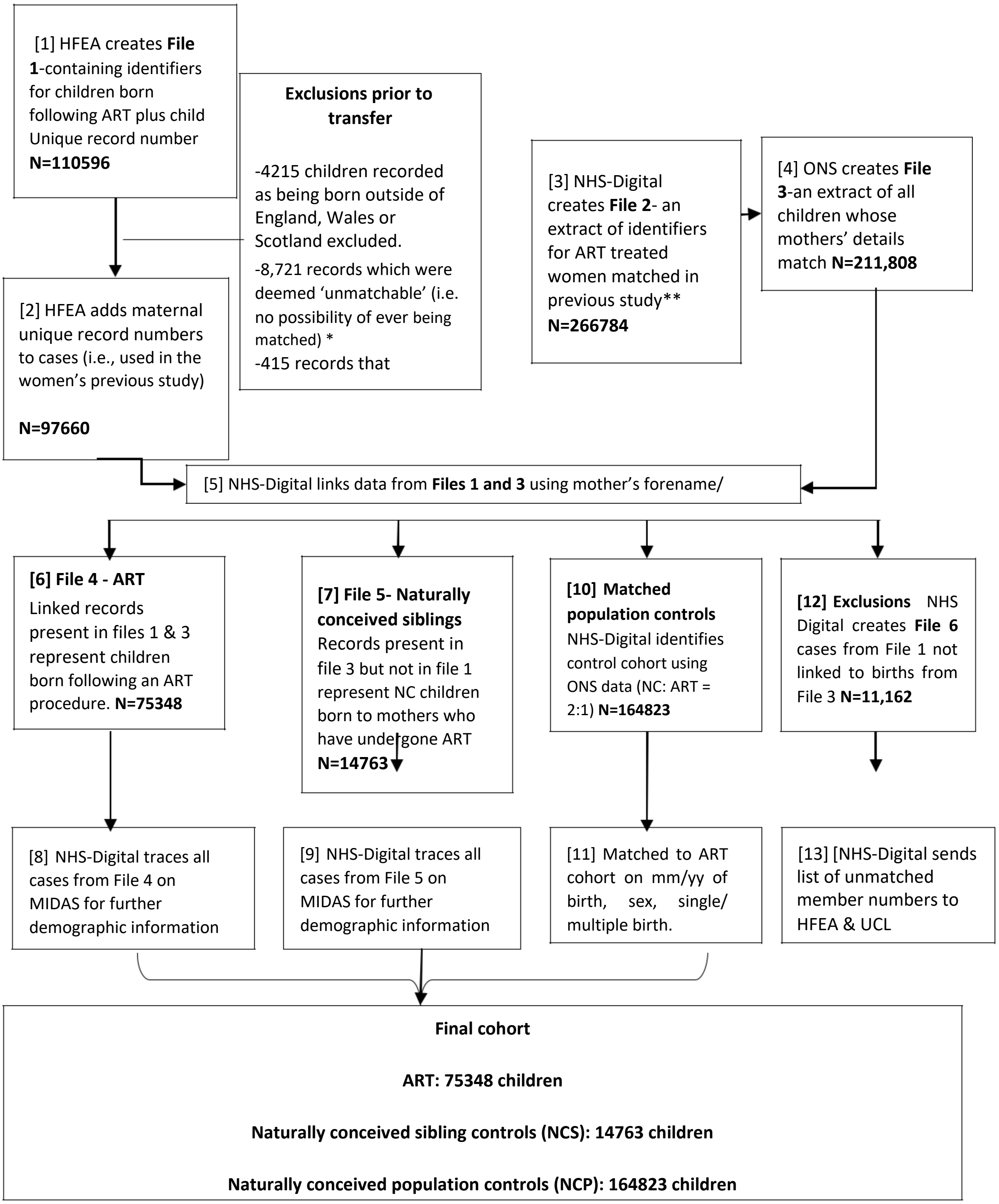

*as they were a) births outside of England/ Wales; b) births before 1993 (when ONS systems were automated and thus the date from which linkage is possible to ONS records); and c) to mothers which were not included in file 2 (as it was not possible to identify them on NHS-Digital systems previously- 'women's study').

** Please see Supplementary figure S2 for cohort flow

HFEA: Human Fertilization and Embryology Authority; ONS: Office for National Statistics; NHS: National Health Service; MIDAS: Medical Integrated Database and Administration System; UCL: University College London; ART: Assisted Reproductive Technology.

Supplementary file S2: Stage 2 of linkage (HFEA-ONS-HES)

[14] NHS Digital produces current status report on all ART, NCS and NCP controls (as produced in linkage 1) and provides de-identified output plus non identifiable deprivation score to UCL.

↓

[15] NHS Digital links cohort data to HES data and provides de-identified output to UCL

↓

[16] UCL interacts with HFEA to obtain fertility treatment clinical data and parental data per matched group member.

UCL match up NHS Digital and clinical HES information to HFEA information using unique record number.

↓

[17] UCL excludes records of children born before 1st April 1997 to coincide with start of HES monitoring.

UCL excludes triplets and higher order births from all groups (along with associated ART, siblings and matched controls)

↓

[18] NHS Digital produces file 4 for each group (identifiable details as below).

- For the ART group, file 4 will be securely transported to the HFEA and stored there securely with highly restricted access.
- For the NCS and NCP groups, file 4 will be securely stored at NHS Digital again with highly restricted access.

↓

Final sub-cohort

HES-ART: 63877

Naturally conceived sibling controls (HES-NCS): 11343

Naturally conceived population controls (HES-NCP): 127544

HFEA: Human Fertilization and Embryology Authority; ONS: Office for National Statistics; NHS: National Health Service; UCL: University College London; ART: Assisted Reproductive Technology; HES: Hospital Episode Statistics database.

Supplementary Table S3: 1CD-10 codes included in the analysis

ICD codes	Description
P00-P04	New-born affected by maternal factors and by complications of pregnancy, labour, and delivery
P05-P08	Disorders of new-born related to length of gestation and fetal growth
P09-P09	Abnormal findings on neonatal screening
P10-P15	Birth trauma
P19-P29	Respiratory and cardiovascular disorders specific to the perinatal period
P35-P39	Infections specific to the perinatal period
P50-P61	Haemorrhagic and haematological disorders of new-born
P70-P74	Transitory endocrine and metabolic disorders specific to new-born
P76-P78	Digestive system disorders of new-born
P80-P83	Conditions involving the integument and temperature regulation of new-born.
P84-P84	Other problems with new-born
P90-P96	Other disorders originating in the perinatal period

Supplementary Table S4: Hospital admissions by diagnosis, sub-cohort, and multiplicity – 2002 to 2009

	ART			NCP			sART			NCS		
	**All (n=44618)**	**Singletons (n=26525)**	**Twins (n=18093)**	**All (n=89072)**	**Singletons (n=52975)**	**Twins (n=36097)**	**All (n=8318)**	**Singletons(n=5686)**	**Twins (n=2632)**	**All (n=8462)**	**Singletons (n=8100)**	**Twins (n=362)**
Any perinatal diagnosis	17,132 (38.39%)	7250 (27.33%)	9882 (54.62%)	30306 (34.02%	10829 (20.44%)	19477 (53.96%)	2716 (32.65%)	1396 (24.55%)	1320 (50.15%)	1738 (20.53%)	1563 (19.30%)	175 (48.34%)
New-borns affected by maternal factors & complications of pregnancy, labour, & delivery	707 (1.58%)	280 (3.86%)	427 (3.94%)	632 (0.71%)	218 (2.21%)	414 (2.13%)	76 (0.91%)	51 (3.65%)	25 (1.89%)	68 (0.80%)	60 (3.84%)	8 (4.57%)
Disorders of new-born related to length of gestation & fetal growth	4931 (11.05%)	1961 (27.05%)	2970 (27.43%)	21273 (23.88%)	6956 (70.39%)	14317 (73.51%)	1263 (15.18%)	379 (27.15%)	884 (66.97%)	487 (5.75%)	371 (23.74%)	116 (66.29%)
Birth trauma	816 (1.82%)	308 (4.25%)	508 (4.69%)	154 (0.17%)	62 (0.63%)	92 (0.47%)	80 (0.96%)	64 (4.58%)	16 (1.21%)	64 (0.75%)	63 (4.03%)	1 (0.57%)
Respiratory & cardiovascular disorders specific to the perinatal period	4226 (9.47%)	1679 (23.16%)	2547 (23.53%)	2194 (2.46%)	746 (7.55%)	1448 (7.43%)	416 (5.00%)	310 (22.21%)	106 (8.03%)	375 (4.43%)	365 (23.35%)	10 (5.71%)
Infections specific to the perinatal period	520 (1.16%)	203 (2.80%)	317 (2.93%)	278 (0.31%)	92 (0.93%)	186 (0.95%)	57 (0.68%)	45 (3.22%)	12 (0.91%)	52 (0.61%)	49 (3.13%)	3 (1.71%)
Haemorrhagic & haematological disorders of new-born	2883 (6.46%)	1256 (17.32%)	1627 (15.02%)	2067 (2.32%)	766 (7.75%)	1301 (6.68%)	347 (4.17%)	224 (16.05%)	123 (9.32%)	256 (3.02%)	241 (15.42%)	15 (8.57%)
Transitory endocrine & metabolic disorders specific to new-born	574 (1.28%)	268 (3.70%)	306 (2.83%)	664 (0.74%)	251 (2.54%)	413 (2.12%)	81 (0.97%)	53 (3.80%)	28 (2.12%)	51 (0.60%)	47 (3.01%)	4 (2.29%)

Digestive system disorders of new-born	105 (0.23%)	35 (0.48%)	70 (0.65%)	145 (0.16%)	52 (0.53%)	93 (0.48%)	15 (0.18%)	10 (0.72%)	5 (0.38%)	18 (0.21%)	17 (1.09%)	1 (0.57%)
Conditions involving the integument & temperature regulation of new-born	839 (1.88%)	307 (4.23%)	532 (4.91%)	366 (0.41%)	152 (1.54%)	214 (1.10%)	86 (1.03%)	67 (4.80%)	19 (1.44%)	115 (1.32%)	115 (7.36%)	0 (0.00%)
Other disorders originating in the perinatal period	1527 (3.42%)	614 (8.47%)	913 (8.43%)	1016 (1.14%)	360 (3.64%)	656 (3.37%)	132 (1.58%)	116 (8.31%)	61 (4.62%)	141 (1.66%)	127 (8.13%)	14 (8.00%)
Missing	951 (2.13%)	339 (4.68%)	612 (5.65%)	570 (0.63%)	227 (2.30%)	343 (1.76%)	118 (1.41%)	77 (5.52%)	41 (3.11%)	111 (1.31%)	108 (6.91%)	3 (1.71%)

ART: Assisted reproductive technology; NCP: Naturally conceived population controls; sART: ART children with NC siblings; NCS: Naturally conceived siblings.

Supplementary Table S5: Risk of perinatal event overall and by chapter

	ART-NCP *adjusted for year of birth, sex, IMD decile, ethnicity, maternal age group at delivery.				sART –NCS *adjusted for sex, year of birth, maternal age group at delivery, birth order [+ family as matching variable]	
	**Singletons**		**Twins**		**Singletons**	
	**RR**	**95% CI**	**RR**	**95% CI**	**RR**	**95% CI**
Any perinatal diagnosis	**1.30**	**1.26, 1.34**	**1.01**	**0.99, 1.03**	**0.97**	**0.84, 1.12**
New-borns affected by maternal factors and complications of pregnancy, labour, & delivery	1.17	0.99, 1.39	1.05	0.88, 1.25	0.98	0.52, 1.83
Disorders of new-born related to length of gestation & fetal growth	1.37	1.29, 1.46	0.99	0.97, 1.01	1.17	0.86, 1.60
Birth trauma	1.23	1.04, 1.44	1.37	0.97, 1.94	0.78	0.47, 1.30
Respiratory & cardiovascular disorders specific to the perinatal period	1.28	1.20, 1.38	0.94	0.86, 1.03	0.72	0.53, 0.98
Infections specific to the perinatal period	1.30	1.06, 1.59	0.98	0.75, 1.27	0.68	0.24, 1.90
Haemorrhagic & haematological disorders of new-born	1.39	1.28, 1.51	1.12	1.02, 1.22	1.02	0.73, 1.44
Transitory endocrine & metabolic disorders specific to new-born	1.34	1.11, 1.61	1.12	0.95, 1.32	1.38	0.61, 3.13
Digestive system disorders of new-born	0.88	0.56, 1.40	1.15	0.80, 1.65	0.77	0.21, 2.79
Conditions involving the integument & temperature regulation of new-born	1.13	0.96, 1.32	1.34	1.07,1.67	0.66	0.35, 1.24
Other disorders originating in the perinatal period	1.35	1.20, 1.52	1.04	0.91, 1.19	1.15	0.48, 2.77
