# Supplementary material for: Perinatal health in a cohort of children conceived after assisted reproduction in the UK: a population-based record-linkage study
Source: BMJ Open. 2024 Nov 11;14(11):e091910. doi: 10.1136/bmjopen-2024-091910 (PMC11555099; doi:10.1136/bmjopen-2024-091910)
Supplement: Supplementary file 2 [file bmjopen-2024-091910.reviewer_comments.pdf]

PEER REVIEW HISTORY

BMJ Open publishes all reviews undertaken for accepted manuscripts. Reviewers are asked to complete a checklist review form and are provided with free text boxes to elaborate on their assessment. These free text comments are reproduced below.

This paper was submitted to another journal from BMJ but declined for publication following peer review. The authors addressed the reviewers' comments and submitted the revised paper to BMJ Open. The paper was subsequently accepted for publication at BMJ Open.

ARTICLE DETAILS

Title (Provisional)

Perinatal health in a cohort of children conceived after assisted reproduction in the UK: a population-based record-linkage study.

Authors

Purkayastha, Mitana; Sutcliffe, Alastair; Brison, Daniel R; Nelson, Scott M; Lawlor, Deborah; Roberts, Stephen A

VERSION 1 - REVIEW

**Reviewer**	**1**
**Name**	**Cavoretto, Paolo**
**Affiliation**	**Univ Vita Salute San Raffaele, Obstetrics and Gynaecology**
**Date**	**03-Apr-2024**
**COI**	**I have no competing interests**

This manuscript reinforces the concept that neonates from IVF /ICSI are at higher risk for a series of severe postnatal issues necessitating hospital admission particularly those related to length of gestation and fetal growth, birth trauma, respiratory and cardiovascular disorders, or infections. The extent of deviation from normal is less pronounced in siblings from the same mother and natural conception.
General Comment
The methods are good, statistical power is remarkable, results are somehow novel and interesting. I have few minor suggestions before publication of this paper. The limitation related to the use of administrative data (with potential risk of bias of unknown degree) should be emphasized a bit more. I recommend also some issues listed below of which importantly emerges the need of reinforcing the clinical

background in relation to the available knowledge on the topic. The clinical translation may be also reinforced.

Specific comments

1. Discussion. There are Important references and points missing in the discussions, and they are highly pertinent to their scopes. Recent data from different research groups showed that uterine artery Doppler pulsatility index in IVF/ICSI pregnancies conceived after frozen-thawed blastocyst transfer, as compared to fresh, have lower uterine artery pulsatility index at 7–37 weeks gestation and a lower rate of SGA suggesting an improved placental perfusion. Similarly, pregnancies conceived with frozen-thawed vs fresh blastocyst transfers present greater crown rump length, estimated fetal weight and neonatal weight with an excess of large for gestational age fetuses and neonates in frozen-thawed conceptions and an excess of small for gestational age and growth restricted fetuses and neonates in fresh conceptions. This overall suggested a greater placental perfusion in pregnancies after thawed transfers, including egg donation due to absence of corporal lutea as compared to pregnancies with fresh embryo transfers (multiple corpora lutea). This issue should be incorporated in the discussion along with major references. Please the authors briefly discuss this essential issue along with major references which are highly pertinent to their results.
2. In relation to the comment above, while it is true what stated by the authors that the overall COPP was similar in fresh and frozen, on the other side in tale 3 emerges clearly that in fresh there is a significant odds ratio of 1.16 for any perinatal diagnosis with an advantage of frozen thawed and this is particularly evident for disorders of fetal growth and this issue brings back attention to comment n 1 (importance of outcomes related to placental function which in this study is confirmed to be probably better in frozen vs fresh).
3. I would suggest adding a brief comment in the discussion with some insight in relation to the evident clinically usefulness and possible clinical translation of these findings in the clinical practice. May these findings be of help in the routine practice? May this knowledge be applied to extend screening methods or can be applied as an aid to diagnostic methods?
4. While it is true that same family sibling reduces the impact of the association to the IVF/ICSI procedure, it is also true that most of these patients are achieving reproduction only trough medically assisted reproduction methods. Therefore, these complications are somehow unavoidable, and these abnormal outcomes may be interpreted as a trade-off between unsuccessful human reproduction (failure) and reproduction achieving a high-risk pregnancy (success). This dichotomy may be considered in the discussion.
5. Another limitation of the study that may be recognized by the authors is the failure to recognize differences among different phenotypes of preterm birth, at least in the fundamental dichotomy between iatrogenic and spontaneous. This may be mentioned in the discussion as there are major evidence showing that both components are more common as compared to spontaneous conception, particularly

the iatrogenic and the outcome, as the authors know, relates substantially to the aetiology of preterm birth.

Minor
Some reference look odd (e.g,.n 3), I am not sure this is the highest quality reference on the tiopic.

**Reviewer**	**2**
**Name**	**Coddington, Charles**
**Affiliation**	**Atrium Health**
**Date**	**03-Apr-2024**
**COI**	**None**

The manuscript entitled "Perinatal health in a cohort of children conceived after assisted reproduction in the UK: a population-based record-linkage study." was read with great interest. The manuscript reviews the Reposts of outcomes for IVF in the UK from their database. The review appears to be very thorough and comprehensive.
The manuscript appears to be well written and proper editorial steps taken.
The only comment that would be germane would be review and inclusion of articles from North America as there might be very reasonable comparisons to broader application. Since the readership is world-wide in would seem appropriate,

**Reviewer**	**3**
**Name**	**Siristatidis, Charalampos.**
**Affiliation**	**National and Kapodistrian University of Athens**
**Date**	**03-Apr-2024**
**COI**	**None**

This is a population-based record-linkage study of children born after assisted reproduction in the United Kingdom between 2002 and 2009. Authors aimed to compare risk of hospitalization for conditions originating in the perinatal period between children conceived via assisted reproductive technology and those that are naturally conceived, differentiating by treatment type.
They concluded that children conceived via assisted reproduction showed modest increases in the risk of hospitalizations for severe perinatal events when compared to population controls, although these findings were attenuated in the sibling analyses.
I believe that the topic that authors are dealing with is very interesting. I have some restrictions though – and some points that should be revisited.

1. Authors should give the aetiology of the specific study period (1997-2009); for example, why not up to 2023? The criteria should be further clarified; the reader should not gain the feeling that authors having a large dataset, could conduct a further study afterwards, using similar or other populations, to answer a related question.
2. Authors should revise their statement on “ART twins (RR 1.01, 95% CI: 0.99, 1.03) exhibited a higher risk of hospital admission for any COPP “.
3. Authors should reconsider their methodology in terms of avoiding the confounding of infertility per se. I am skeptical when differences are lost in the secondary analyses, while the latter is limited, as authors correctly describe in their limitations section.

**Reviewer**	**4**
**Name**	**Wang, Rui**
**Affiliation**	**Monash University, Department of Obstetrics and Gynaecology**
**Date**	**03-Apr-2024**
**COI**	**None**

Purkayastha and colleagues conducted a population-based record-linkage study to compare the risk of hospitalisation due to perinatal complications in neonates conceived via ART and naturally conceived neonates. They found that neonates conceived via ART had modest increases in the risk of hospitalizations compared to naturally conceived neonates, and that these findings were attenuated in the sibling analyses.
The strength of the study includes the use of a sibling design and the consideration of different types of ART. The limitations include the cohort identification and linkage, the period of recruitment of the study, as well as the relatively broad definition of perinatal outcomes.
The authors may wish to consider the following comments:
1. Neonates selection process: the authors presented two supplementary figures to illustrate the two stages of the overall data linkage, but did not present the selection process that led to the number of neonates included in the current study. Can the authors present a flowchart that clearly shows the selection process (including the numbers of neonates conceived via different subtypes of ART)?
2. Although the focus of the study is perinatal health, the inclusion of only neonates requiring hospitalisation during the first 7 days of life ignores the most important perinatal outcomes including stillbirth and neonatal death. Would it be possible to report these outcomes at all?
3. For all analyses of twin neonates, it seems that the cluster effect within twin siblings was not considered in the analysis (at least based on the methods

described). The authors are encouraged to address this in the analyses of twin siblings.
4. It would be useful to present the absolute event rates alongside the risk ratios for all outcomes as the event rates could be rare for multiple outcomes, especially in the sub-analyses on types of ART.
5. Different denominators are used in Table 2, which is a bit confusing. It would be recommended to report the event rates across the table as per comment #4 above.
6. The statement on the main findings (modest overall association and attenuated association in the sibling analyses) does not capture all scenarios. There are a couple of examples where the directions of associations are even opposite in the overall analysis and the sibling analysis among singletons and the confidence intervals in both analyses do not overlap (e.g. respiratory & cardiovascular disorders). The authors are encouraged to address and discuss these findings.
7. How was missing data handled and what is the rationale for choosing this strategy to handle missing data?
8. The authors published another study on childhood outcomes with similar comparisons (Sutcliffe et al. AJOG. 2023). It is worth noting that some observed perinatal outcomes do not translate to subsequent childhood outcomes. Similariy, some observed differences in childhood outcomes are not reflected in perinatal outcomes. It would be useful to have some discussions to reflect on these findings at different stages during childhood development.
9. As the study period is between 2002 and 2009, it would be useful to address the main differences of ART in terms of indications and technologies to help readers interpret the findings in the context of practice changes.

**Reviewer**	**5**
**Name**	**Perera, Rafael**
**Affiliation**	**University of Oxford**
**Date**	**03-Apr-2024**
**COI**	**None**

The current manuscript presents a series of analysis aiming quantify the relative risk of adverse prenatal outcomes from assisted reproduction compared to normal birth. To do this, the authors use two comparator groups. The first is a matched cohort using year of birth, gender, and parity as matching variables. The second is a within family comparison, using a normal birth sibling as control.

Their main findings are an excess risk in the overall perinatal events and in some of the individual events for the matched cohort but not for the sibling comparison. This is roughly in line with previous research which has shown a protective effect when compared to siblings.

There are multiple inaccuracies in the reporting and some specific questions about the way the cohorts were created.

1. The rate of events is extremely high. This rate is similar to that reported for Ethiopia (doi: 10.1136/bmjopen-2021-053881). Although it is unclear if it is the same outcome. Please can you check what is the natural baseline rate in a non-selected population to check for potential biases?

2. The flow charts provided give total numbers for the different cohorts that do not match the results or the abstract. Please correct.

3. The results in percentages reported in table 2 are for the events, not the population. Please change to population as otherwise the comparisons do not make sense. Some of these percentages are wrong.

4. The current explanationand conclusion that more data would have given the right answer is not credible. More data will reduce confidence intervals. The current point estimates for the comparisons in the two controls are on either side of the null. That is, they are not consistent. Please adjust your wording overall. The explanation of this discrepancy given in the discussion section appears valid. More information about how to account for this bias would have been beneficial.

5. The adjustments done are having somemajor impact on the estimates. The raw rates appear in opposition tothe model findings. This could be due to some severe imbalances in the groups. For example the difference is socio economic status and maternal age. A further discussion of thisand exploration is needed.

6. Please provide more information about how you handle multiple children (twins) in your model.

7. Finally, the reporting of some estimates as effective while the 95%CI include the null is not valid. Please adjust.

VERSION 1 - AUTHOR RESPONSE

Reviewer 1: Dr. Paolo Cavoretto, IRCCS Ospedale San Raffaele

This manuscript reinforces the concept that neonates from IVF /ICSI are at higher risk for a series of severe postnatal issues necessitating hospital admission particularly those related to length of gestation and fetal growth, birth trauma,

respiratory and cardiovascular disorders, or infections. The extent of deviation from normal is less pronounced in siblings from the same mother and natural conception.

General comment

The methods are good, statistical power is remarkable, results are somehow novel and interesting. I have few minor suggestions before publication of this paper. The limitation related to the use of administrative data (with potential risk of bias of unknown degree) should be emphasized a bit more. I recommend also some issues listed below of which importantly emerges the need of reinforcing the clinical background in relation to the available knowledge on the topic. The clinical translation may be also reinforced.

Response: Thank you for your valuable feedback. The following text has now been included in the manuscript:

Page 19 Lines 314-316: *Moreover, although HES data have been used extensively for research purposes, there have been long-standing concerns regarding the quality, completeness and coverage of records within health services and the academic community.*

Specific comment

Comment 1
Discussion. There are Important references and points missing in the discussions, and they are highly pertinent to their scopes. Recent data from different research groups showed that uterine artery Doppler pulsatility index in IVF/ICSI pregnancies conceived after frozen-thawed blastocyst transfer, as compared to fresh, have lower uterine artery pulsatility index at 7–37 weeks gestation and a lower rate of SGA suggesting an improved placental perfusion. Similarly, pregnancies conceived with frozen-thawed vs fresh blastocyst transfers present greater crown rump length, estimated fetal weight and neonatal weight with an excess of large for gestational age fetuses and neonates in frozen-thawed conceptions and an excess of small for gestational age and growth restricted fetuses and neonates in fresh conceptions. This overall suggested a greater placental perfusion in pregnancies after thawed transfers, including egg donation due to absence of corporal lutea as compared to pregnancies with fresh embryo transfers (multiple corpora lutea). This issue should be incorporated in the discussion along with major references. Please the authors briefly discuss this essential issue along with major references which are highly pertinent to their results.

Response: Thank you very much for your valuable feedback. The Discussion section of the manuscript has now been revised to include the suggested information along with pertinent references.

Comment 2
In relation to the comment above, while it is true what stated by the authors that the overall COPP was similar in fresh and frozen, on the other side in tale 3 emerges clearly that in fresh there is a significant odds ratio of 1.16 for any perinatal diagnosis with an advantage of frozen thawed and this is particularly evident for disorders of fetal growth and this issue brings back attention to comment n 1 (importance of outcomes related to placental function which in this study is confirmed to be probably better in frozen vs fresh).

Response: Thank you very much for your valuable feedback. The Discussion section of the manuscript has now been revised to include the suggested information along with pertinent references.

Comment 3
I would suggest adding a brief comment in the discussion with some insight in relation to the evident clinically usefulness and possible clinical translation of these findings in the clinical practice. May these findings be of help in the routine practice? May this knowledge be applied to extend screening methods or can be applied as an aid to diagnostic methods?

Response: Thank you for your valuable feedback. The following text has now been included in the manuscript:

<u>Page 20 Lines 333-336:</u> *Nevertheless, the findings of this study provide vital insight into the health of children born after ART, potentially facilitating identification of at-risk individuals/families; contextualization of levels and trends of disease burden and hospitalization; informing affected patients and their families and also existing health services; allow for wider health system resource planning and anticipation of future health resource needs; and contribute to the development of effective care pathways.*

Comment 4
While it is true that same family sibling reduces the impact of the association to the IVF/ICSI procedure, it is also true that most of these patients are achieving reproduction only trough medically assisted reproduction methods. Therefore, these complications are somehow unavoidable, and these abnormal outcomes may be interpreted as a trade-off between unsuccessful human reproduction (failure) and reproduction achieving a high-risk pregnancy (success). This dichotomy may be considered in the discussion.

Response: Thank you for your valuable comment. The Discussion section has now been revised to reflect this.

Comment 5
Another limitation of the study that may be recognized by the authors is the failure to recognize differences among different phenotypes of preterm birth, at least in the

fundamental dichotomy between iatrogenic and spontaneous. This may be mentioned in the discussion as there are major evidence showing that both components are more common as compared to spontaneous conception, particularly the iatrogenic and the outcome, as the authors know, relates substantially to the aetiology of preterm birth.

Response: Thank you for your valuable comment. The Discussion section has now been revised to reflect this.

Comment 6
Some reference look odd (e.g,.n 3), I am not sure this is the highest quality reference on the topic.

Response: Thank you. The following reference has now been included in the manuscript:

Page 23: *[4] Nelson SM, Lawlor DA. Predicting Live Birth, Preterm Delivery, and Low Birth Weight in Infants Born from In Vitro Fertilisation: A Prospective Study of 144,018 Treatment Cycles. PLOS Medicine. 2011;8(1):e1000386.*

Reviewer 2: Dr. Charles Coddington, Carolinas HealthCare System

Comment 1

The manuscript entitled "Perinatal health in a cohort of children conceived after assisted reproduction in the UK: a population-based record-linkage study." was read with great interest. The manuscript reviews the Reposts of outcomes for IVF in the UK from their database. The review appears to be very thorough and comprehensive.

The manuscript appears to be well written and proper editorial steps taken.

The only comment that would be germane would be review and inclusion of articles from North America as there might be very reasonable comparisons to broader application. Since the readership is world-wide in would seem appropriate,

Response: Thank you. Prior to manuscript preparation, we carried out a thorough systematic search of current evidence with the aim of including high-quality studies conducted in various populations. No restrictions on country of publication were applied and the final list of references includes several systematic reviews and meta-analyses, thereby ensuring adequate coverage of global evidence.

Reviewer 3: Dr. C. Siristatidis, Aretaie Univ Hosp

This is a population-based record-linkage study of children born after assisted reproduction in the United Kingdom between 2002 and 2009. Authors aimed to compare risk of hospitalization for conditions originating in the perinatal period between children conceived via assisted reproductive technology and those that are naturally conceived, differentiating by treatment type.
They concluded that children conceived via assisted reproduction showed modest increases in the risk of hospitalizations for severe perinatal events when compared to population controls, although these findings were attenuated in the sibling analyses.
I believe that the topic that authors are dealing with is very interesting. I have some restrictions though – and some points that should be revisited.

Comment 1
Authors should give the aetiology of the specific study period (1997-2009); for example, why not up to 2023? The criteria should be further clarified; the reader should not gain the feeling that authors having a large dataset, could conduct a further study afterwards, using similar or other populations, to answer a related question.

Response: Thank you for your valuable comment. The specific study period used here was selected based on the quality of data available prior to 2002 as well as a change in HFEA legislation implemented in 2009 which sought to actively collect consent from patients. As a result, the cohort post-2009 is not only limited in terms of coverage but also has potential for significant bias. A detailed description of this has been provided under the Study inclusion and exclusion criteria section (shown below). Additionally, a new flowchart showing creation of the study sub-cohort (Figure 1) has also been included on Page 7 of the manuscript (also shown below).

Page 6 Lines 123-138: *The current study focused on a sub-group of the original study cohort born between 2002 and 2009 only. A lower limit of 2002 was applied as the NHS Numbers for Babies (NN4B) service was introduced in this year, allowing maternity staff in England to request an NHS number for babies in hospital after birth using an on-line system as part of the Statutory Birth Notification process. Prior to this, babies were allocated NHS numbers by registrars at birth registration which could take up to 6 weeks. Previous studies exploring the impact of changes to data collection over time on coverage and completeness of linked follow-up records for children reported unreliable linkages between births and follow-up records before 2002, evidenced by underestimation of mortality and hospital re-admission rates.*

An upper limit of 2009 was applied in keeping with HFEA legislation. Consent for disclosure of information for research was not collected from patients who underwent treatment at a licensed fertility clinic prior to September 2009 (although consent could be withdrawn retrospectively), thus permitting linkage of these individuals (and any children born thereafter) to other datasets. However, since 1st October 2009,

prospective consent for research use of data has been made mandatory and low overall consent rates which varied between fertility clinics has cast doubt on the validity of research conducted using HFEA register data recorded after this date.

Figure 1: Flowchart showing creation of study cohort.

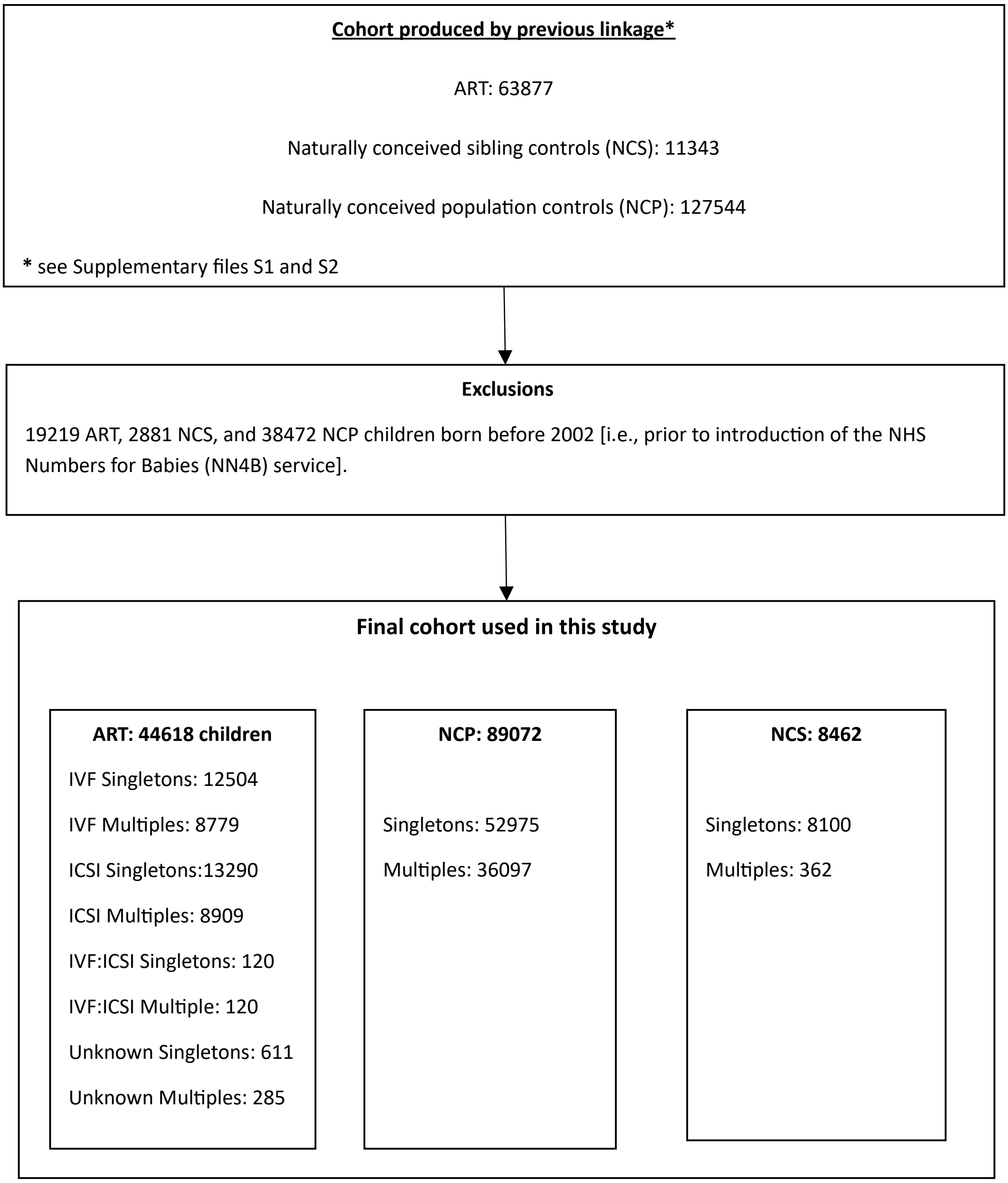

ART: Assisted reproductive technology; NCP: Naturally conceived population controls; NCS: Naturally conceived siblings; IVF: In-vitro fertilization; ICSI: Intracytoplasmic sperm injection

Comment 2
Authors should revise their statement on "ART twins (RR 1.01, 95% CI: 0.99, 1.03) exhibited a higher risk of hospital admission for any COPP ".

Response: Thank you for your comment. The text has now been revised as follows:

Page 11 Lines 194-197

Original text: Both ART singletons (RR: 1.30, 95% CI: 1.26, 1.34) and ART twins (RR 1.01, 95% CI: 0.99, 1.03) exhibited a higher risk of hospital admission for any COPP when compared to the corresponding matched NCP sub-cohorts (Figure 1 & Supplementary Table S4).

Revised text: Both ART singletons (RR: 1.30, 95% CI: 1.26, 1.34) and ART twins (RR 1.01, 95% CI: 0.99, 1.03) exhibited directionally similar increases in risk of hospital admission for any COPP when compared to the corresponding matched NCP sub-cohorts, although the latter was not statistically significant (Figure 2 & Supplementary Table S4).

Comment 3
Authors should reconsider their methodology in terms of avoiding the confounding of infertility per se. I am skeptical when differences are lost in the secondary analyses, while the latter is limited, as authors correctly describe in their limitations section.

Response:

Thank you for your valuable comment.

Conventional multivariable approaches to explore the association of ART with adverse perinatal outcomes are essential in order to extrapolate effect sizes and risk estimates to the general ART population; however, they cannot fully account for parental factors related to sub-fertility which may confound these associations. In contrast, comparison of siblings born after different conception methods offers an opportunity to disentangle the contributions of factors such as ART treatment, shared genetics, parental health factors, confounding from background family socioeconomic position etc.; however, these analyses are often limited by lower statistical power due to restricted numbers, a higher risk of bias if there is individual level confounding, and the possibility that the subset of ART children with siblings may not be representative of the ART population as a whole

Therefore, we believe that a key strength of our study is the use of both approaches, with one minimizing selection bias and the other minimizing confounding. Furthermore, all analyses carried out were pre-specified in an analysis plan that was subject to wide expert review prior to commencement of the study. Although the findings observed in the ART vs NCP comparison were lost in the within-sibling analysis, the inherent limitations of the NCS cohort and the statistical inefficiency of sibling analyses in general prevented us from making robust conclusions on whether these results were different to the ART-NCP results. We believe that this suggests

that the findings of the within-family analysis must be interpreted with caution in this case, and does not necessarily discredit the underlying methodology which has several strengths including increased confidence when the findings are similar in both groups and the ability to control for unmeasured maternal and family characteristics which are more likely to be similar within women than between women.

Reviewer 4: Dr. Rui Wang, Monash University

Purkayastha and colleagues conducted a population-based record-linkage study to compare the risk of hospitalisation due to perinatal complications in neonates conceived via ART and naturally conceived neonates. They found that neonates conceived via ART had modest increases in the risk of hospitalizations compared to naturally conceived neonates, and that these findings were attenuated in the sibling analyses.
The strength of the study includes the use of a sibling design and the consideration of different types of ART. The limitations include the cohort identification and linkage, the period of recruitment of the study, as well as the relatively broad definition of perinatal outcomes.
The authors may wish to consider the following comments:

Comment 1
Neonates selection process: the authors presented two supplementary figures to illustrate the two stages of the overall data linkage, but did not present the selection process that led to the number of neonates included in the current study. Can the authors present a flowchart that clearly shows the selection process (including the numbers of neonates conceived via different subtypes of ART)?

Response: Thank you for your comment. We have now included a flowchart (Figure 1) showing the cohort selection process including number of neonates by subtypes of ART and multiplicity on Page 7 of the manuscript (please also see Reviewer 3 comment 1 above).

Comment 2
Although the focus of the study is perinatal health, the inclusion of only neonates requiring hospitalisation during the first 7 days of life ignores the most important perinatal outcomes including stillbirth and neonatal death. Would it be possible to report these outcomes at all?

Response: Thank you for your valuable suggestion. The current study linked the cohorts to hospital data only, preventing examination of stillbirths and neonatal deaths accurately. However, this can potentially be addressed in a future study aiming to link this existing cohort to mortality data.

Comment 3
For all analyses of twin neonates, it seems that the cluster effect within twin siblings was not considered in the analysis (at least based on the methods described). The authors are encouraged to address this in the analyses of twin siblings.

Response: The ART vs NCS family-matched models included a family covariate as strata to allow for within-family correlations. The text has now been revised to clarify this further, as follows:

<u>Original text:</u> For comparisons of sART vs NCS, similar models were fitted adjusting for year of birth; maternal age at delivery; sex; and order of pregnancy (grouped into

1st, 2nd, and >2nd) with an additional family covariate (fixed effect) to allow for within-family correlations.

Revised text: For comparisons of sART vs NCS, a maternal id cluster was used to create family-matched models adjusted for year of birth; maternal age at delivery; sex; and order of pregnancy (grouped into 1st, 2nd, and >2nd) to allow for within-family correlations. IMD and ethnicity were not included as the underlying effects they represent would have remained constant within families.

Comment 4
It would be useful to present the absolute event rates alongside the risk ratios for all outcomes as the event rates could be rare for multiple outcomes, especially in the sub-analyses on types of ART.

Response: Thank you for your valuable feedback. Table 3 has now been revised to include the number of events (please see below and page 15 of the manuscript) by treatment type. It was not possible to include the RRs and 95% CI's in Table 2 due to its size and the event rates reported here should be interpreted in conjunction with figure 2 and supplementary table S4.

Table 3: Event rate and risk of hospitalization for any and diagnosis-specific perinatal events by ART treatment type

	IVF (n=21283)	ICSI (n=22199)	ICSI vs IVF		Fresh embryo transfer (n=38964)	Frozen embryo transfer (n=5620)	Fresh vs Frozen embryo transfer	
	**No. of events (%)**	**No. of events (%)**	**RR**	**95% CI**	**No. of events (%)**	**No. of events (%)**	**RR**	**95% CI**
Any perinatal diagnosis	**8287 (38.93%)**	**8442 (38.02%)**	**0.96**	**0.94, 0.98**	**15228 (39.08%)**	**1888 (33.5%)**	**1.16**	**1.12, 1.20**
New-borns affected by maternal factors and complications of pregnancy, labour, & delivery	365 (3.47%)	326 (3.49%)	1.10	0.95, 1.29	468 (3.07%)	67 (3.55%)	0.96	0.77, 1.20
Disorders of new-born related to length of gestation & fetal growth	5754 (54.68%)	4921 (52.69%)	0.91	0.88, 0.94	8388 (55.08%)	829 (43.91%)	1.42	1.34, 1.51
Birth trauma	238 (2.26%)	199 (2.13%)	0.89	0.74, 1.08	329 (2.16%)	52 (2.75%)	0.91	0.70, 1.19
Respiratory & cardiovascular disorders specific to the perinatal period	1387 (13.18%)	1359 (14.55%)	1.10	1.03, 1.19	2011 (13.21%)	301 (15.94%)	0.99	0.89, 1.10
Infections specific to the perinatal period	157 (1.49%)	156 (1.67%)	1.12	0.89, 1.40	226 (1.48%)	46 (2.44%)	0.73	0.55, 0.98
Haemorrhagic & haematological disorders of new-born	1186 (11.27%)	1065 (11.40%)	0.99	0.91, 1.08	1671 (10.97%)	276 (14.62%)	0.87	0.78, 0.98
Transitory endocrine & metabolic disorders specific to new-born	261 (2.48%)	279 (2.99%)	1.18	1.00, 1.40	412 (2.71%)	52 (2.75%)	1.17	0.90, 1.51
Digestive system disorders of new-born	41 (0.39%)	42 (0.45%)	1.05	0.68, 1.62	67 (0.44%)	6 (0.32%)	1.38	0.67, 2.87
Conditions involving the integument & temperature regulation of new-born	260 (2.47%)	237 (2.54%)	1.01	0.85, 1.21	386 (2.53%)	46 (2.44%)	1.10	0.84, 1.45
Other disorders originating in the perinatal period	526 (5.00%)	453 (4.85%)	0.91	0.80, 1.03	804 (5.28)	113 (5.99%)	1.03	0.86, 1.24
Missing	349 (3.32%)	303 (3.24%)	-	-	466 (3.06%)	100 (5.30%)	-	-

ART: Assisted reproductive technology; RR: Risk Ratio; CI: Confidence interval; ICSI: Intracytoplasmic sperm injection; IVF: In-vitro fertilization

Comment 5
Different denominators are used in Table 2, which is a bit confusing. It would be recommended to report the event rates across the table as per comment #4 above.

Response: Thank you for your valuable feedback. Table 2 has now been revised to include event rates within each cohort overall and stratified by multiplicity (please see revised table below and on page 12 of manuscript) to allow appropriate comparisons.

Table 2: Hospital admissions by diagnosis, sub-cohort, and multiplicity – 2002-2009

	ART			NCP			sART			NCS		
	**All (n=44618)**	**Singletons (n=26525)**	**Twins (n=18093)**	**All (n=89072)**	**Singletons (n=52975)**	**Twins (n=36097)**	**All (n=8318)**	**Singletons (n=5686)**	**Twins (n=2632)**	**All (n=8462)**	**Singletons (n=8100)**	**Twins (n=362)**
Any perinatal diagnosis	17,132 (38.39%)	7250 (27.33%)	9882 (54.62%)	30306 (34.02%	10829 (20.44%)	19477 (53.96%)	2716 (32.65%)	1396 (24.55%)	1320 (50.15%)	1738 (20.53%)	1563 (19.30%)	175 (48.34%)
New-borns affected by maternal factors and complications of pregnancy, labour, & delivery	707 (1.58%)	280 (3.86%)	427 (3.94%)	632 (0.71%)	218 (2.21%)	414 (2.13%)	76 (0.91%)	51 (3.65%)	25 (1.89%)	68 (0.80%)	60 (3.84%)	8 (4.57%)
Disorders of new-born related to length of gestation & fetal growth	4931 (11.05%)	1961 (27.05%)	2970 (27.43%)	21273 (23.88%)	6956 (70.39%)	14317 (73.51%)	1263 (15.18%)	379 (27.15%)	884 (66.97%)	487 (5.75%)	371 (23.74%)	116 (66.29%)
Birth trauma	816 (1.82%)	308 (4.25%)	508 (4.69%)	154 (0.17%)	62 (0.63%)	92 (0.47%)	80 (0.96%)	64 (4.58%)	16 (1.21%)	64 (0.75%)	63 (4.03%)	1 (0.57%)
Respiratory & cardiovascular disorders specific to the perinatal period	4226 (9.47%)	1679 (23.16%)	2547 (23.53%)	2194 (2.46%)	746 (7.55%)	1448 (7.43%)	416 (5.00%)	310 (22.21%)	106 (8.03%)	375 (4.43%)	365 (23.35%)	10 (5.71%)
Infections specific to the perinatal period	520 (1.16%)	203 (2.80%)	317 (2.93%)	278 (0.31%)	92 (0.93%)	186 (0.95%)	57 (0.68%)	45 (3.22%)	12 (0.91%)	52 (0.61%)	49 (3.13%)	3 (1.71%)
Haemorrhagic & haematological disorders of new-born	2883 (6.46%)	1256 (17.32%)	1627 (15.02%)	2067 (2.32%)	766 (7.75%)	1301 (6.68%)	347 (4.17%)	224 (16.05%)	123 (9.32%)	256 (3.02%)	241 (15.42%)	15 (8.57%)
Transitory endocrine & metabolic disorders specific to new-born	574 (1.28%)	268 (3.70%)	306 (2.83%)	664 (0.74%)	251 (2.54%)	413 (2.12%)	81 (0.97%)	53 (3.80%)	28 (2.12%)	51 (0.60%)	47 (3.01%)	4 (2.29%)
Digestive system disorders of new-born	105 (0.23%)	35 (0.48%)	70 (0.65%)	145 (0.16%)	52 (0.53%)	93 (0.48%)	15 (0.18%)	10 (0.72%)	5 (0.38%)	18 (0.21%)	17 (1.09%)	1 (0.57%)

Conditions involving the integument & temperature regulation of new-born	839 (1.88%)	307 (4.23%)	532 (4.91%)	366 (0.41%)	152 (1.54%)	214 (1.10%)	86 (1.03%)	67 (4.80%)	19 (1.44%)	115 (1.32%)	115 (7.36%)	0 (0.00%)
Other disorders originating in the perinatal period	1527 (3.42%)	614 (8.47%)	913 (8.43%)	1016 (1.14%)	360 (3.64%)	656 (3.37%)	132 (1.58%)	116 (8.31%)	61 (4.62%)	141 (1.66%)	127 (8.13%)	14 (8.00%)
Missing	951 (2.13%)	339 (4.68%)	612 (5.65%)	570 (0.63%)	227 (2.30%)	343 (1.76%)	118 (1.41%)	77 (5.52%)	41 (3.11%)	111 (1.31%)	108 (6.91%)	3 (1.71%)

Comment 6
The statement on the main findings (modest overall association and attenuated association in the sibling analyses) does not capture all scenarios. There are a couple of examples where the directions of associations are even opposite in the overall analysis and the sibling analysis among singletons and the confidence intervals in both analyses do not overlap (e.g. respiratory & cardiovascular disorders). The authors are encouraged to address and discuss these findings.

Response: Thank you for your valuable feedback. As acknowledged in lines 251-254 and 300-302 of the manuscript, the overall sample size of our NCS cohort was limited which, in turn, resulted in fairly small numbers for certain diagnostic groups (as reflected in the wide confidence intervals). Therefore, we have refrained from discussing the findings of these individual groups specifically as these limitations prevented us from drawing robust conclusions on whether these results were different to those observed in the ART-NCP analysis.

The following text has now been revised in the manuscript to reflect this:

<u>Page 16 Lines 251-254 Original text:</u> Although this might be interpreted as suggesting that confounding, including by parental causes of infertility may explain the observed population control association, we acknowledge that the sibling sample size was relatively small and that the wide confidence intervals, particularly for specific conditions, meant we could not robustly claim these results were different to the ART-NCP results.

<u>Revised text:</u> Although this might be interpreted as suggesting that confounding, including by parental causes of infertility may explain the observed population control association, we acknowledge that the sibling sample size was relatively small and that the wide confidence intervals, particularly for specific conditions, meant we could not robustly claim these results were different to the ART-NCP results. Therefore, the findings of the within-family analysis must be interpreted with caution, and the differences in estimates for the individual diagnostic groups between the ART-NCP and sART-NCS comparisons warrants further exploration.

<u>Page 18 Lines 324-327 Original text:</u> The current study showed modest increases in the risk of hospital admissions for any COPP among ART-conceived children when compared to NCP controls, with attenuation to the null of these findings within siblings.

<u>Revised text:</u> The current study showed modest increases in the risk of hospital admissions for any COPP among ART-conceived children when compared to NCP controls, with attenuation to the null of these findings within siblings. However, the findings of the latter should be interpreted with caution due to the limited sample size.

Comment 7
How was missing data handled and what is the rationale for choosing this strategy to handle missing data?

Response: Thank you. We have previously reported the details of how missing data was handled at the linkage stage in our publication titled ‘Cohort profile: a national, population-based cohort of children born after assisted conception in the UK (1992–2009): methodology and birthweight analysis’ (doi: 10.1136/bmjopen-2021-050931). Although journal restrictions on word count prevented us from including this information in the current manuscript, we have referenced this previous publication abundantly and urge the reader to refer to it for further clarity on the creation of the underlying cohort.

Comment 8

The authors published another study on childhood outcomes with similar comparisons (Sutcliffe et al. AJOG. 2023). It is worth noting that some observed perinatal outcomes do not translate to subsequent childhood outcomes. Similarly, some observed differences in childhood outcomes are not reflected in perinatal outcomes. It would be useful to have some discussions to reflect on these findings at different stages during childhood development.

Response: Thank you, we do acknowledge that some early outcomes may not necessarily have lasting effects, and further studies involving larger ART populations and longer follow-up are necessary to elucidate late effects on factors such as childhood growth patterns, risk of subtle neurodevelopmental issues, risk of premature ageing etc.

Comment 9

As the study period is between 2002 and 2009, it would be useful to address the main differences of ART in terms of indications and technologies to help readers interpret the findings in the context of practice changes.

Response: Thank you. The two major changes in treatment practices in the post-2009 era are (1) a greatly increased use of single embryo transfers with a concomitant reduction in dizygotic twinning (from >30% to below 10% now), and (2) a greatly increased use of extended embryo culture to blastocyst stage prior to fresh transfer. However, a lack of data post-2009 prevented us from exploring the influence of changes in ART treatment practices in the current study and we have no acknowledged this as a limitation of the study and have added the following text to the manuscript:

<u>Page 19 Lines 308-315:</u> *. Rapid advances in ART technologies in the last 10 years, particularly greatly increased use of single embryo transfers with a concomitant reduction in dizygotic twinning and a rise in the use of extended embryo culture to blastocyst stage prior to fresh transfer, emphasizes the urgent need to continue to prospectively monitor the next cohort of children from 2010 onwards. Although some studies have explored the influence of these changes as well as others (e.g., timelapse incubators, changes in stimulation protocols etc.), the lack of data post-2009 limited our ability to explore the effects of changes in ART techniques over time and draw potential inferences in relation to current ART conceptions.(46)*

Reviewer 5: Prof. Rafael Perera, University of Oxford

The current manuscript presents a series of analysis aiming quantify the relative risk of adverse prenatal outcomes from assisted reproduction compared to normal birth. To do this, the authors use two comparator groups. The first is a matched cohort using year of birth, gender, and parity as matching variables. The second is a within family comparison, using a normal birth sibling as control.

Their main findings are an excess risk in the overall perinatal events and in some of the individual events for the matched cohort but not for the sibling comparison. This is roughly in line with previous research which has shown a protective effect when compared to siblings.

There are multiple inaccuracies in the reporting and some specific questions about the way the cohorts were created.

Comment 1
The rate of events is extremely high. This rate is similar to that reported for Ethiopia (doi: 10.1136/bmjopen-2021-053881). Although it is unclear if it is the same outcome. Please can you check what is the natural baseline rate in a non-selected population to check for potential biases?

Response: Thank you for your valuable comment. The study conducted by Jaleta et al., (doi: 10.1136/bmjopen-2021-053881) examined predictors of adverse perinatal outcomes in 777 women who gave birth at a single centre in Ethiopia. The authors reported that the incidence of adverse perinatal outcomes in this cohort was approximately 31.5% (i.e., n= ~245 women). In contrast, the current study estimated risk of hospitalisation for adverse perinatal outcomes in children conceived via ART. Therefore, the differences in the underlying populations of the two studies prevent comparison of the event rates.

Comment 2
The flow charts provided give total numbers for the different cohorts that do not match the results or the abstract. Please correct.

Response: Thank you for your comment. The flowcharts shown in the Supplementary files represent the various stages of the original linkage carried out. The current study used a sub-cohort (2002-2009) of this original linkage and these are the numbers that have been reported in the Abstract and Results sections. The specific study period used here was selected based on the quality of data available prior to 2002 as well as HFEA legislation which limited data availability after 2009. A detailed description of this has been provided under the Study inclusion and exclusion criteria section (shown below). Additionally, a new flowchart showing creation of the study sub-cohort (Figure 1) has also been included on Page 7 of the manuscript (please also see Reviewer 3 comment 1 above).

Page 6 Lines 123-138: *The current study focused on a sub-group of the original study cohort born between 2002 and 2009 only. A lower limit of 2002 was applied as the NHS Numbers for Babies (NN4B) service was introduced in this year, allowing maternity staff in England to request an NHS number for babies in hospital after birth using an on-line system as part of the Statutory Birth Notification process. Prior to this, babies were allocated NHS numbers by registrars at birth registration which could take up to 6 weeks. Previous studies exploring the impact of changes to data collection over time on coverage and completeness of linked follow-up records for children reported unreliable linkages between births and follow-up records before 2002, evidenced by underestimation of mortality and hospital re-admission rates.*

An upper limit of 2009 was applied in keeping with HFEA legislation. Consent for disclosure of information for research was not collected from patients who underwent treatment at a licensed fertility clinic prior to September 2009 (although consent could be withdrawn retrospectively), thus permitting linkage of these individuals (and any children born thereafter) to other datasets. However, since 1st October 2009, prospective consent for research use of data has been made mandatory and low overall consent rates which varied between fertility clinics has cast doubt on the validity of research conducted using HFEA register data recorded after this date.

Comment 3
The results in percentages reported in table 2 are for the events, not the population. Please change to population as otherwise the comparisons do not make sense. Some of these percentages are wrong.

Response: Thank you for your valuable feedback. Table 2 has now been revised to include event rates within each cohort overall and stratified by multiplicity (please see revised table above and on page 12 of manuscript) to allow appropriate comparisons.

Comment 4
The current explanation and conclusion that more data would have given the right answer is not credible. More data will reduce confidence intervals. The current point estimates for the comparisons in the two controls are on either side of the null. That is, they are not consistent. Please adjust your wording overall. The explanation of this discrepancy given in the discussion section appears valid. More information about how to account for this bias would have been beneficial.

Response: Thank you for your valuable comment. Although we do highlight the small sample size and statistical inefficiency of the sibling analysis as a limitation of the current study, we believe that more data would not only reduce the confidence intervals but also give more precise point estimates, thereby allowing us to determine if they are or are not consistent with each other and different from zero. The Discussion has now been revised to reflect this.

Comment 5
The adjustments done are having some major impact on the estimates. The raw rates appear in opposition to the model findings. This could be due to some severe imbalances in the groups. For example the difference is socio economic status and maternal age. A further discussion of this and exploration is needed.

Response: Thank you for your valuable comment. The groups are expected to differ in both measured and unmeasured characteristics. In the current study, we have adjusted for the measured characteristics and, as the reviewer says, these adjustments do make a difference. The unadjusted results are shown for completeness purposes only and do not represent a comparison of interest in themselves and, therefore, we do not believe are worthy of discussion.

Comment 6
Please provide more information about how you handle multiple children (twins) in your model.

Response: The ART vs NCS family-matched models included a family covariate as strata to allow for within-family correlations. The text has now been revised to clarify this further, as follows:

<u>Original text:</u> For comparisons of sART vs NCS, similar models were fitted adjusting for year of birth; maternal age at delivery; sex; and order of pregnancy (grouped into 1st, 2nd, and >2nd) with an additional family covariate (fixed effect) to allow for within-family correlations.

<u>Revised text:</u> For comparisons of sART vs NCS, a maternal id cluster was used to create family-matched models adjusted for year of birth; maternal age at delivery; sex; and order of pregnancy (grouped into 1st, 2nd, and >2nd) to allow for within-family correlations. IMD and ethnicity were not included as the underlying effects they represent would have remained constant within families.

Comment 7
Finally, the reporting of some estimates as effective while the 95%CI include the null is not valid. Please adjust.

Response: Thank you. The following changes have been made to the manuscript:

<u>Page 11 Lines 194-197 Original text:</u> Both ART singletons (RR: 1.30, 95% CI: 1.26, 1.34) and ART twins (RR 1.01, 95% CI: 0.99, 1.03) exhibited a higher risk of hospital admission for any COPP when compared to the corresponding matched NCP sub-cohorts (Figure 1 & Supplementary Table S4).

<u>Revised text:</u> Both ART singletons (RR: 1.30, 95% CI: 1.26, 1.34) and ART twins (RR 1.01, 95% CI: 0.99, 1.03) exhibited directionally similar increases in risk of hospital admission for any COPP when compared to the corresponding matched NCP sub-cohorts, although the latter was not statistically significant (Figure 2 & Supplementary Table S4).

VERSION 2 - REVIEW

**Reviewer**	**1**
**Name**	**Cavoretto, Paolo**
**Affiliation**	**Univ Vita Salute San Raffaele, Obstetrics and Gynaecology**
**Date**	**07-Sep-2024**
**COI**	**I have no competing interests**

I believe that most issues presented in my first review were resolved or clarified. The article is now ready for publication.

**Reviewer**	**2**
**Name**	**Wang, Rui**
**Affiliation**	**Monash University, Department of Obstetrics and Gynaecology**
**Date**	**19-Sep-2024**
**COI**	**NA**

I would like to thank the authors for providing this excellent revision. I only have one minor remark

In the abstract, the authors stated "This risk was attenuated in the within-sibling analysis [0.97 (0.84, 1.12)]. Similar patterns were seen for ... respiratory & cardiovascular disorders [vs population controls: 1.28 (1.20, 1.38); vs siblings: 0.72 (0.53, 0.98)];". I acknowledge that the authors would not like to discuss individual findings. It would be great if the authors could rephrase the sentence, as "attenuated" does not seem to be accurate for all scenarios.

VERSION 2 - AUTHOR RESPONSE

Reviewer 1: Dr. Paolo Cavoretto, Univ Vita Salute San Raffaele

I believe that most issues presented in my first review were resolved or clarified. The article is now ready for publication.

Response: Thank you very much for your valuable feedback which has allowed us to improve our manuscript considerably.

Reviewer 2: Dr. Rui Wang, Monash University

I would like to thank the authors for providing this excellent revision. I only have one minor remark
In the abstract, the authors stated "This risk was attenuated in the within-sibling analysis [0.97 (0.84, 1.12)]. Similar patterns were seen for ... respiratory & cardiovascular disorders [vs population controls: 1.28 (1.20, 1.38); vs siblings: 0.72 (0.53, 0.98)];". I acknowledge that the authors would not like to discuss individual findings. It would be great if the authors could rephrase the sentence, as "attenuated" does not seem to be accurate for all scenarios.

Response: Thank you very much for your valuable feedback. We have now revised the text to reflect this, as follows:

Original text: *This risk was attenuated in the within-sibling analysis [0.97 (0.84, 1.12)]. Similar patterns were seen for diagnoses related to length of gestation and fetal growth [vs population controls: 1.37 (1.29, 1.46); vs siblings: 1.17 (0.86, 1.60)]; birth trauma (vs population controls: 1.23 (1.04, 1.44); vs siblings: 0.78 (0.47, 1.30)]; respiratory & cardiovascular disorders [vs population controls: 1.28 (1.20, 1.38); vs siblings: 0.72 (0.53, 0.98)]; infections [vs population controls: 1.30 (1.06, 1.59); vs siblings: 0,68 (0.24, 1.90)] and several other conditions.*

Revised text: *However, no such increase was observed in the within-sibling analysis [0.97 (0.84, 1.12)]. Similar patterns were seen for diagnoses related to length of gestation and fetal growth [vs population controls: 1.37 (1.29, 1.46); vs siblings: 1.17 (0.86, 1.60)]; birth trauma (vs population controls: 1.23 (1.04, 1.44); vs siblings: 0.78 (0.47, 1.30)]; respiratory & cardiovascular disorders [vs population controls: 1.28 (1.20, 1.38); vs siblings: 0.72 (0.53, 0.98)]; infections [vs population controls: 1.30 (1.06, 1.59); vs siblings: 0,68 (0.24, 1.90)] and several other conditions.*
